# Clarifying the Scope of Native EHRA Type 2 Valvular Disease and Event Reporting in Anticoagulated Patients With Atrial Fibrillation

**DOI:** 10.1002/clc.70358

**Published:** 2026-05-28

**Authors:** Jiaqi Chen, Mangmang Xu

**Affiliations:** ^1^ The Second Clinical Medicine College of Zhejiang Chinese Medical University Hangzhou China

To the Editor

We read with great interest the article by Escolar Conesa et al. which evaluated the prognostic implications of native valvular heart disease in anticoagulated patients with atrial fibrillation using the EHRA valvular heart disease classification [1]. The authors should be commended for addressing an important and clinically relevant question: whether patients traditionally grouped under “non‐valvular” atrial fibrillation but with native valvular involvement represent a distinct higher‐risk population [2]. In a cohort of 1399 anticoagulated patients, the authors reported that 886 patients were classified as EHRA type 2 with native valve disease and 513 as EHRA type 3 without valve disease. Over a median follow‐up of 910 days, the native EHRA type 2 group had higher rates of major bleeding, all‐cause mortality, cardiovascular mortality, heart failure, MACE, and net clinical outcome, and native EHRA type 2 status remained independently associated with several adverse outcomes in multivariable analyzes. These findings are clinically meaningful and support closer attention to valvular status in the risk assessment of patients with atrial fibrillation.

Nevertheless, several issues may benefit from further clarification to improve the interpretability and clinical applicability of the findings.

First, the distinction between the formal EHRA type 2 category and the population actually analyzed in this study deserves more explicit clarification. According to the EHRA classification described by the authors, type 2 valvular heart disease includes not only native valvular involvement but also biological prostheses and previous valve interventions such as transcatheter aortic valve replacement and MitraClip [3]. However, the study explicitly excluded patients with any type of surgical or percutaneous prosthesis. Therefore, the analyzed EHRA type 2 group appears to represent a narrower subgroup of patients with native valvular disease rather than the full EHRA type 2 population. This distinction is clinically important because patients with native valve disease, biological prostheses, transcatheter valve interventions, or mitral repair devices may differ substantially in baseline risk, valve‐related hemodynamics, antithrombotic management, bleeding risk, thromboembolic risk, and long‐term prognosis. Consequently, although this study provides valuable evidence that native valvular involvement is associated with worse outcomes among anticoagulated patients with atrial fibrillation, its conclusions should be framed more precisely as applying to “native EHRA type 2” patients rather than to the entire EHRA type 2 category. Clearer terminology would strengthen the interpretability of the findings and avoid potential overgeneralization of the prognostic implications of the EHRA type 2 classification.

Second, the presentation of event percentages in Table 3 may also require clarification. Although the authors state that the percentages are calculated with respect to the total cohort, the table is organized by EHRA group, and therefore, readers may reasonably interpret the values in parentheses as group‐specific cumulative event rates. For example, major bleeding is reported as 82 events (5.9%) in the native EHRA type 2 group and 22 events (1.6%) in the EHRA type 3 group. However, these percentages appear to use the overall cohort of 1399 patients as the denominator, rather than the corresponding group sizes of 886 and 513. When calculated within each EHRA group, the event proportions would be approximately 9.3% and 4.3%, respectively. Similar differences apply to other outcomes: all‐cause mortality would be approximately 23.9% versus 11.5%, heart failure 36.6% versus 11.5%, MACE 22.3% versus 8.4%, and net clinical outcome 33.4% versus 17.7% for native EHRA type 2 and EHRA type 3 patients, respectively. These recalculated proportions do not contradict the authors' main conclusion; rather, they reinforce the higher absolute burden of adverse events in native EHRA type 2 patients. Because the main purpose of Table 3 is to compare adverse event burden between EHRA categories, reporting group‐specific event proportions alongside incidence rates per 100 patient‐years and hazard ratios would improve clinical interpretability and reduce the possibility that readers underestimate or misinterpret the absolute risk within each EHRA group.

Third, the statistical modeling strategy would benefit from additional methodological detail, as it directly affects the interpretation of the reported independent association between native EHRA type 2 status and adverse outcomes. In the Methods section, the authors refer to “univariate and multivariate Cox logistic regression analysis,” whereas the results are presented as hazard ratios and the study includes time‐to‐event outcomes, suggesting that Cox proportional hazards models were used rather than logistic regression. This terminology should be clarified because Cox regression and logistic regression address fundamentally different outcome structures. More importantly, the manuscript does not report whether the proportional hazards assumption was assessed, how covariates were selected for the multivariable models, whether collinearity among closely related clinical variables and risk scores was considered, or how missing data were handled. In addition, given the advanced age and substantial mortality burden of the cohort, competing risks may be particularly relevant for non‐fatal outcomes such as stroke, major bleeding, and heart failure [4]. The handling of composite endpoints, including whether MACE and net clinical outcome were analyzed as time‐to‐first events, also deserves clarification. Providing these details would strengthen the transparency and robustness of the statistical analysis and would allow readers to better judge whether native EHRA type 2 status is an independent prognostic marker beyond differences in age, comorbidity burden, anticoagulation quality, and baseline cardiovascular risk.

In summary, this study makes an important contribution by highlighting the adverse prognostic profile of anticoagulated patients with atrial fibrillation and native valvular disease. The above clarifications would not weaken the central message of the article; rather, they would refine the scope of inference, improve the transparency of absolute risk reporting, and enhance confidence in the statistical interpretation of the findings. Such refinements may further support the use of valve status as a meaningful clinical marker in the risk stratification of patients with atrial fibrillation.

## Conflicts of Interest

The authors declare no conflicts of interest.

## Data Availability

The data that support the findings of this study are available from the corresponding author upon reasonable request.
